# Trends in incidence and outcomes of revision total hip arthroplasty in Spain: A population based study

**DOI:** 10.1186/1471-2474-13-37

**Published:** 2012-03-19

**Authors:** Manuel Villanueva-Martınez, Valentın Hernandez-Barrera, Francisco Chana-Rodríguez, José Rojo-Manaute, Antonio Rıos-Luna, Jesus San Roman Montero, Angel Gil-de-Miguel, Rodrigo Jimenez-Garcıa

**Affiliations:** 1 Orthopedic Department, Hospital Gregorio Marañon, Madrid, Spain; 2 Preventive Medicine and Public Health Teaching and Research Unit, Department of Health Sciences, Universidad Rey Juan Carlos, Madrid, Spain; 3 Orthopedic Department, Virgen del Mar Hospital, Almeria, Spain

**Keywords:** Revision, Hip arthroplasty, Cost, Mortality, Outcome research, Osteoarthritis, Revision, Hospital, Charlson Index, Discharge Database

## Abstract

**Background:**

To analyze changes in incidence and outcomes of patients undergoing revision total hip arthroplasty (RTHA) over an 8-year study period in Spain.

**Methods:**

We selected all surgical admissions in individuals aged ≥ 40 years who underwent RTHA (ICD-9-CM procedure code 81.53) between 2001 and 2008 from the Spanish National Hospital Discharge Database. Age- and sex-specific incidence rates, Charlson co-morbidity index, length of stay (LOS), costs and in-hospital mortality (IHM) were estimated for each year. Multivariate analyses were conducted to asses time trends.

**Results:**

32, 280 discharges of patients (13, 391 men/18, 889 women) having undergone RTHA were identified. Overall crude incidence showed a small but significant increase from 20.2 to 21.8 RTHA per 100, 000 inhabitants from 2001 to 2008 (p < 0.01).

The incidence increased for men (17.7 to 19.8 in 2008) but did not vary for women (22.3 in 2001 and 22.2 in 2008). Greater increments were observed in patients older than 84 years and in the age group 75-84. In 2001, 19% of RTHA patients had a Charlson Index ≥ 1 and this proportion rose to 24.6% in 2008 (p < 0.001). The ratio RTHA/THA remained stable and around 20% in Spain along the entire period

The crude overall in-hospital mortality (IHM) increased from 1.16% in 2001 to 1.77% (p = 0.025) in 2008. For both sexes the risk of death was higher with age, with the highest mortality rates found among those aged 85 or over. After multivariate analysis no change was observed in IHM over time. The mean inflation adjusted cost per patient increased by 78.3%, from 9, 375 to 16, 715 Euros from 2001 to 2008.

After controlling for possible confounders using Poisson regression models, we observed that the incidence of RTHA hospitalizations significantly increased for men and women over the period 2001 to 2008 (IRR 1.10, 95% CI 1.03-1.18 and 1.08, 95% CI 1.02-1.14 respectively).

**Conclusions:**

The crude incidence of RTHA in Spain showed a small but significant increase from 2001 to 2008 with concomitant reductions in LOS, significant increase in co-morbidities and cost per patient.

## Background

Although recent reports based on National Arthroplasty Registries show that the overall 10-year survival of total hip arthroplasty (THA) is over 90%, the burden of Revision Total Hip Arthroplasty (RTHA) is growing in developed countries [1,2]. Patients undergoing RTHA usually suffer from several co-morbidities, technical difficulties and complications requiring higher resource utilization than THA [3-5].

Surveys from several countries have reported a continued growth in the use of THA and RTHA over the last decades as a result of ageing populations, the extension of indications and of the age range for this treatment [3-5].

In addition, the results and functional improvement of RTHA seems to be inferior to THA with greater length of hospital stay (LOS) and higher cost [6-9].

Comparisons of primary and revision rates and outcomes between countries may provide information that would help for understanding the differences as well as aid for planning the provision of healthcare services [10].

Unfortunately Spain does not have a national arthroplasty registry. In the absence of such a registry, the discharge databases can provide a large alternative information source to describe and analyze the trends and characteristics of THA and RTHA at a national level [11,12].

The aim of this study was to analyze national representative data, collected through the Spanish Hospital Discharge Database [13] from 2001 to 2008 to elucidate changes in incidence; demographic characteristics; co-morbidity profiles; LOS; costs; and, in-hospital mortality (IHM) of patients undergoing RTHA.

## Methods

According to the Spanish Health System Organization, each physician must declare at the time of discharge all diagnoses and procedures performed for each hospitalization using the code of the International Classification of Disease, 9th revision (ICD-9CM). This information is collected by the Spanish National Hospital Database, namely "Conjunto Mínimo Básico de Datos" (CMBD), which compiles all the public and private hospital data and covers more than 95% of hospital discharges [14]. The CMBD database includes patients' variables (sex, date of birth), date of admittance, date of discharge, discharge destination (home, deceased or other health/social institution), up to 14 discharge diagnosis and up to 20 procedures performed during the admission. Social or health institutions include nursing homes and long-term care medical centers.

In Spain since 1999 the National Statistics Institute has used the ICD-10 to codify the underlying cause of death. But the CMBD has not been changed to the 10th revision and the ICD-9 cm is still being used [13,14].

We included all surgical admissions (elective or emergency admissions) in patients 40 years or over, who received a RTHA procedure (ICD-9-CM procedure code 81.53) during 2001-2008. We calculated the yearly age- and sex-specific incidence rates by dividing the number of RTHA cases per year per sex and age group by the corresponding population per group according to the National Institute of Statistics (INE), reported on December 31^st ^each year [15]. Incidence rates were expressed per 100.000 inhabitants. The proportion of patients that died during hospital admission (IHM), LOS, and costs was also estimated for each year studied using the number of RTHA as the denominator. Costs were calculated using Diagnosis-Related Groups (DRG) for the disease. DRG represents a medical-economic entity concerning a set of diseases requiring analogous management resources [16]. All costs shown are adjusted for inflation over the same period in Spain according to the National Institute for Statistics [17]. Spain has a universal public health system so every person legally residing in or visiting Spain receives all medical and surgical treatment free of charge. As in most European countries, the reimbursing system is based on the DRG.

Clinical characteristics included information in overall co-morbidity at the time of surgery, which was assessed by computing the Charlson comorbidity index (CCI) [18]. The CCI is not a tool routinely used in all hospital patients, so we calculated the CCI for each patient based on coded data available at the discharge register. We divided patients into 3 categories: low index, which corresponded to patients with no previously recorded disease categories in the CCI; medium index, patients with one or two disease categories; and high index, patients with more than two disease categories. We used these three cutting points, even though it is not the classification suggested by literature, to make our results comparable to other reports from Spain and to a previous study on primary total hip arthroplasty using the same methods [5,19].

We also analyzed if the total number of RTHA changed in relation to the number of Primary THA performed by calculating the ratio RTHA/THA per year and the time trend.

### Statistical analysis

Quantitative variables were expressed as means, medians, range and inter-quartile range (IQR). Qualitative variables were expressed as frequencies and percentages. Comparisons were done using the chi-square test, Fisher's exact test, Student's t-test, Wilcoxon rank-sum test, ANOVA or Kruskal-Wallis test as appropriate. The multivariate analysis for time trends in the variables studied was conducted using Poisson, lineal and logistic regression models adjusted by age, sex and other co-variables when appropriate. Estimations were made using STATA program and statistical significance was set at α < 0·05 (two-tailed).

The cumulated and anonymised data were delivered by the Ministry of Health, the official institute holding the Spanish National Hospital Database. Thus, data protection was fully guaranteed. Given the anonymous and mandatory nature of the data, the requirement for informed consent was not necessary.

## Results

The Spanish population changed over the study period. According to the Spanish National Statistics Institute [15], in 2001 the population living in Spain was 41.12 million; by 2008 the population it had increased to 46.16 million. The proportion of immigrant population increased from 3.33% in 2001 to 11.42% in 2008. As a consequence of this mainly young immigrant population, the proportion of people aged 65 or over decreased from 17.2% to 16.54% in this period.

**Figure 1:**
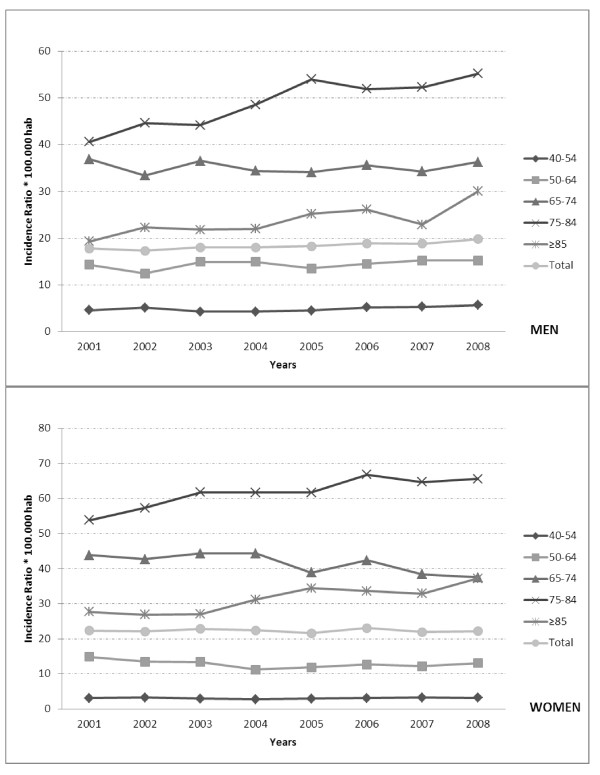
**Incidence of RTHA per 100, 000 inhabitants in Spain from 2001 to 2008, according to age group and gender**.

From 2001 to 2008, we identified a total of 32, 280 patient discharges (13, 391 men and 18, 889 women) that underwent a RTHA. Table 1 and Figure 1 displays the total numbers and the incidence of RTHA per 100, 000 inhabitants in each year and according to age group and gender.

**Table 1:** Age and sex-specific incidence rates for RTHA and total number of RTHA and primary THA in Spain (2001-8)

		2001	2002	2003	2004	2005	2006	2007	2008	
Age		Rate/100, 000	Rate/100, 000	Rate/100, 000	Rate/100, 000	Rate/100, 000	Rate/100, 000	Rate/100, 000	Rate/100, 000	P*
40-54	Men	4.6	5.1	4.3	4.3	4.5	5.2	5.3	5.7	0.007
	Women	3.1	3.3	2.9	2.8	2.9	3.1	3.3	3.2	0.540
50-64	Men	14.3	12.4	14.9	14.9	13.5	14.5	15.2	15.2	0.100
	Women	14.9	13.5	13.4	11.2	11.9	12.7	12.2	13.1	0.054
65-74	Men	36.9	33.4	36.5	34.4	34.1	35.6	34.3	36.3	0.862
	Women	43.8	42.7	44.3	44.3	38.9	42.4	38.4	37.5	< 0.001
75-84	Men	40.6	44.6	44.2	48.6	54.0	51.9	52.3	55.2	< 0.001
	Women	53.8	57.3	61.8	61.7	61.7	66.8	64.7	65.6	< 0.001
≥ 85	Men	19.3	22.3	21.8	22.0	25.2	26.1	22.9	30.0	< 0.001
	Women	27.7	26.9	27.0	31.2	34.4	33.6	32.9	37.2	< 0.001
Total	Men	17.8	17.3	18.0	18.0	18.3	18.9	18.8	19.8	0.107
	Women	22.3	22.1	22.8	22.4	21.6	23.1	21.9	22.2	< 0.001
	Both	20.2	19.9	20.6	20.4	20.1	21.1	20.4	21.1	0.016
Number of RTHA		3, 713	3, 699	3, 912	3, 964	3, 992	4, 294	4, 242	4, 464	
Number of primary THA		18, 185	19, 029	19, 545	19, 733	20, 461	21, 176	21, 354	22, 308	
Ratio RTHA/THA %		20.42	19.44	20.02	20.09	19.51	20.28	19.87	20.01	0.077

Incidence rates (number of cases per year sex and age group/corresponding number of person in that population group that year)

*P value for time trend estimated using Poisson regression models

The overall crude incidence increased from 20.2 RTHA to 21.1 RTHA per 100, 000 inhabitants from 2001 to 2008 (p < 0.001). The incidence of RTHA hospitalizations has increased during the eight year study period for men (17.8 in 2001 to 19.8 in 2008) and not changed for women (22.3 in 2001 and 22.2 in 2008).

Among men, incidence significantly increased for those aged 40-54, 75-84 and ≥ 85 years. Among women, an increase was found among those aged 75-84 and over 84 years, and a decrease was found in the 65-74 years category. The highest incidence for both sexes was found in the 75-84 years category, followed by the 65-74 years category and the 85 or older category. However, the greater increments were observed in the sub-groups of patients older than 84 years and the sub-group 75-84 years.

Table 1 also shows the total number of RTHA and THA and the ratio RTHA/THA per year. As we can see, the percentages remained stable and around 20% with no change overtime.

Time trends in the discharge destinations and CCI for RTHA and primary THA are summarized in Table 2. The results for primary THA are also shown.

**Table 2:** Percentages of discharge destinations and Charlson comorbidity index of RTHA and primary THA patients hospitalized in Spain (2001-2008)

			2001	2002	2003	2004	2005	2006	2007	2008	P*
RTHA	Discharge destinations*	Home	96.0	95.2	95.5	94.5	94.4	94.3	94.8	93.6	< 0.001
		Health/social institution)	4.0	4.8	4.5	5.5	5.6	5.7	5.2	6.4	
	Charlson comorbidity index	Low	81.0	79.7	78.1	77.5	75.7	76.9	75.9	75.4	< 0.001
		Medium	17.8	19.2	20.5	21.3	22.8	21.5	22.5	22.1	
		High	1.2	1.1	1.4	1.2	1.5	1.6	1.6	2.5	
Primary THA	Discharge destinations*	Home	96.8	97.0	96.4	96.4	96.1	95.7	95.9	95.3	< 0.001
		Health/social institution)	3.2	3.0	3.6	3.6	3.9	4.3	4.1	4.7	
	Charlson comorbidity index	Low	81.0	81.2	79.9	79.4	79.2	78.7	77.9	78.5	< 0.001
		Medium	18.4	18.1	19.3	19.9	19.9	20.4	21.3	20.4	
		High	0.6	0.7	0.8	0.7	0.9	0.9	0.8	1.1	

*P value for time trend estimated using logistic regression adjusted by age and sex.

The proportion of individuals with RTHA who were discharged to a social or health institution rose significantly from 4% in 2001 to 6.4% in 2008 (p < 0.001).

In 2001, 81% of RTHA patients had a Charlson Index of 0, 17.8% of 1-2, and in 1.2% it was greater than two. In 2008, the proportion of patients who underwent a RTHA and had a Charlson Index of 1-2 or > 2 increased to 22.1% and 2.5% respectively (p < 0.001). Figures for CCI were very similar for revision and primary THA, however, there were more RTHA than primary THA patients discharged to a Health/social institution.

The mean LOS for RTHA admissions was 20.6 days in 2001 and showed a small but significant decrease to 19.1 days in 2008 (p < 0.01). A decreasing time trend in the LOS was observed in both sexes, Table 3.

**Table 3:** Length of stay in days and hospital costs per patient for RTHA hospitalizations in Spain (2001-2008)

			Year								
			2001	2002	2003	2004	2005	2006	2007	2008	p-value
Length of stay in days	Men	Mean	18.90	19.74	19.56	18.67	19.38	18.19	17.28	17.17	< 0.001
		Median	15.00	14.00	14.00	13.00	13.00	13.00	12.00	12.00	
		IQR	12.00	12.00	13.00	13.00	13.00	11.00	10.00	11.00	
	Women	Mean	21.81	20.80	20.57	19.99	20.68	20.16	19.81	20.53	0.015
		Median	16.00	15.00	15.00	14.00	14.00	14.00	13.00	14.00	
		IQR	14.00	14.00	14.00	14.00	15.00	14.00	13.00	14.00	
	Both	Mean	20.64	20.37	20.17	19.45	20.14	19.35	18.74	19.07	< 0.001
		Median	15.00	15.00	14.00	14.00	14.00	14.00	13.00	13.00	
		IQR	12.00	14.00	14.00	13.00	14.00	13.00	13.00	14.00	
Costs per patient (Euros)	Men	Mean	8, 565.91	9, 857.04	11, 038.01	11, 645.21	11, 663.85	15, 221.23	14, 944.87	15, 011.78	< 0.001
		Median	6, 597.13	7, 256.20	7, 887.54	8, 228.87	7, 800.66	10, 779.02	11, 161.12	9, 986.17	
		IQR	4, 837.90	6, 344.27	7, 109.21	7, 641.10	, 688.20	9, 880.77	8, 370.84	9, 986.17	
	Women	Mean	9, 923.12	10, 291.09	11, 508.02	12, 584.07	12, 334.93	16, 812.99	17, 186.13	18, 029.63	< 0.001
		Median	7, 036.94	7, 320.32	8, 450.94	8, 319.59	8, 400.71	12, 575.53	12, 091.21	11, 650.53	
		IQR	6, 299.19	7, 320.32	7, 601.91	8, 590.88	9, 000.76	11, 095.98	11, 226.38	12, 482.71	
	Both	Mean	9, 375.56	10, 116.96	11, 318.55	12, 200.62	12, 051.34	16, 153.89	16, 232.99	16, 715.40	< 0.001
		Median	6, 914.72	7, 320.32	7, 988.47	8, 228.87	8, 400.71	11, 677.27	11, 206.75	11, 030.62	
		IQR	5, 717.52	6, 832.29	7, 409.53	8, 228.87	8, 400.71	9, 880.77	10, 379.35	11, 646.23	

P value for time trend estimated using logistic and lineal regression models.

IQR Inter quartile range

During the study period, total costs for RTHA increased from 34.8 million to 74.6 million euros, however, the average costs per RTHA increased by 78.3%, from 9, 375 to 16, 715 euros. Women had higher mean and total costs per RTHA than men trough all the years analyzed (Table 3).

IHM rate trends according to sex- and age-groups are shown in Table 4. The risk of death was higher with age for both sexes. The overall IHM rate per RTHA increased from 1.16% in 2001 to 1.77% (p = 0.025) in 2008 (1.4% in women and 0.8% in men in 2001 to 2.02% in women and 1.44% in men in 2008). The highest mortality rates were found among women and men aged 85 or over, nevertheless, there was a significant reduction in male mortality rates in this older group (7.7% in 2001 and 3.5% in 2008) as compared to women (7.8% in 2001 and 10.7% in 2008).

**Table 4:** In-Hospital Mortality, shown as percentage with standard error, after RTHA in Spain (2001-2008) according to age group and sex

		2001	2002	2003	2004	2005	2006	2007	2008	p
Age		(SE)	(SE)	(SE)	(SE)	(SE)	(SE)	(SE)	(SE)	
40-54	Men	0.00 (0.00)	0.00 (0.00)	0.58 (0.01)	0.00 (0.00)	0.00 (0.00)	0.00 (0.00)	0.00 (0.00)	0.00 (0.00)	0, 473
	Women	1.67 (1.17)	0.00 (0.00)	0.00 (0.00)	0.00 (0.00)	0.00 (0.00)	0.72 (0.72)	0.66 (0.66)	0.67 (0.66)	0, 938
55-64	Men	0.00 (0.00)	0.82 (0.58)	0.67 (0.47)	0.00 (0.00)	0.35 (0.35)	0.00 (0.00)	0.00 (0.00)	1.45 (0.64)	0, 281
	Women	0.32 (0.32)	0.00 (0.00)	0.00 (0.00)	0.41 (0.41)	0.00 (0.00)	0.68 (0.48)	0.00 (0.00)	0.63 (0.44)	0, 324
65-74	Men	0.94 (0.38)	0.51 (0.27)	0.47 (0.27)	1.00 (0.41)	0.68 (0.34)	0.49 (0.28)	0.86 (0.38)	1.14 (0.43)	0, 546
	Women	0.55 (0.25)	0.23 (0.19)	0.33 (0.19)	0.66 (0.27)	0.63 (0.28)	0.47 (0.23)	0.78 (0.32)	0.81 (0.33)	0, 146
75-84	Men	0.82 (0.47)	1.48 (0.67)	1.90 (0.67)	1.45 (0.54)	2.52 (0.66)	2.16 (0.62)	2.25 (0.62)	2.07 (0.57)	0, 101
	Women	1.73 (0.48)	1.65 (0.48)	2.05 (0.48)	1.76 (0.44)	1.18 (0.35)	1.15 (0.33)	0.97 (0.30)	1.58 (0.38)	0, 141
≥ 85	Men	7.69 (4.27)	8.33 (3.99)	8.33 (3.99)	10.00 (4.24)	13.33 (4.39)	3.03 (2.11)	11.29 (4.02)	3.45 (1.96)	0, 347
	Women	7.75 (2.35)	6.06 (1.26)	2.21 (1.26)	6.79 (1.98)	2.15 (1.06)	3.68 (1.37)	4.59 (1.50)	10.68 (2.02)	0, 246
Total	Men	0.80 (0.23)	1.01 (0.27)	1.14 (0.27)	1.11 (0.26)	1.60 (0.31)	0.96 (0.23)	1.39 (0.28)	1.44 (0.27)	0, 073
	Women	1.40 (0.25)	1.04 (0.21)	1.03 (0.21)	1.45 (0.25)	0.87 (0.19)	1.03 (0.20)	1.07 (0.21)	2.02 (0.28)	0, 151
	Total	1.16 (0.18)	1.03 (0.16)	1.07 (0.16)	1.31 (0.18)	1.18 (0.17)	1.00 (0.15)	1.20 (0.17)	1.77 (0.20)	0, 025

In-Hospital Mortality (IHM) (number of patients that died during the hospitalization/number of patients' hospitalized)

SE Standard error

*P value for time trend estimated using logistic regression

As can be seen in Table 5 after controlling for possible confounders (age, sex and CCI), using Poisson regression models, we observed that the incidence of RTHA hospitalizations has significantly increased from 2001 to 2008 for both men and women (IRR 1.10, 95% CI 1.03-1.18 and 1.08, 95% CI 1.02-1.14 respectively).

**Table 5:** Multivariate analysis of trends and factors associated with incidence and in-hospital deaths after RTHA

		INCIDENCE OF HOSPITALIZATIONS FOR RTHA			IN-HOSPITAL DEATHS AFTER HOSPITALIZATIONS FOR RTHA		
		Risk Rate Ratios (95% Confidence Intervals)*			Odds Ratios (95% Confidence Intervals)		
		Men	Women	Both	Men	Women	Both
Age	40-54	-	-	-	-	-	-
	55-64	**2.92 (2.75-3.11)**	**4.07 (3.79-4.38)**	**3.36 (3.21-3.52)**	6.35 (0.81-49.61)	0.59 (0.18-1.95)	1.54 (0.60-3.95)
	65-74	**7.14 (6.75-7.55)**	**13.20 (12.37-14.09)**	**9.52 (9.13-9.92)**	**11.00 (1.51-80.22)**	1.24 (0.48-3.18)	**2.88 (1.25-6.65)**
	75-84	**10.02 (9.46-10.61)**	**19.61 (18.38-20.91)**	**13.89 (13.32-14.48)**	**24.89 (3.46-179.24)**	**3.47 (1.40-8.56)**	**7.54 (3.32-17.08)**
	≥ 85	**3.58 (3.23-3.97)**	**11.95 (11.03-12.95)**	**7.18 (6.76-7.61)**	**85.48 (11.69-625.13)**	**13.52 (5.41-33.76)**	**29.90 (13.03-68.60)**
Sex	Men	NA	NA	-	NA	NA	-
	Women	NA	NA	**1.13 (1.10-1.15)**	NA	NA	0.95 (0.77-1.17)
Charlson comorbidity index	0	-	-	-	-	-	-
	1-2	**0.32 (0.30-0.33)**	**0.24 (0.23-0.25)**	**0.27 (0.26-0.28)**	**3.24 (2.28-4.59)**	**3.87 (2.94-5.10)**	**3.69 (2.96-4.58)**
	> 2	**0.028 (0.02-0.03)**	**0.015 (0.01-0.02)**	**0.021 (0.01-0.02)**	**13.98 (9.00-21.73)**	**15.66 (9.53-25.75)**	**17.81 (12.69-24.99)**
YEAR	2001	-	-	-	-	-	-
	2002	0.98 (0.91-1.05)	1.00 (0.94-1.06)	0.99 (0.95-1.04)	1.26 (0.59-2.70)	0.71 (0.41-1.23)	0.85 (0.54-1.34)
	2003	1.02 (0.95-1.09)	1.04 (0.98-1.10)	1.03 (0.98-1.08)	1.15 (0.55-2.40)	0.69 (0.40-1.20)	0.83 (0.53-1.28)
	2004	1.02 (0.95-1.10)	1.02 (0.96-1.08)	1.02 (0.98-1.07)	1.16 (0.56-2.40)	0.91 (0.55-1.51)	0.97 (0.64-1.48)
	2005	1.06 (0.99-1.13)	0.99 (0.93-1.04)	1.01 (0.97-1.06)	1.55 (0.78-3.07)	**0.50 (0.28-0.88)**	0.81 (0.53-1.25)
	2006	**1.08 (1.01-1.16)**	**1.06 (1.00-1.12)**	**1.07 (1.02-1.12)**	1.02 (0.49-2.15)	**0.56 (0.32-0.95)**	0.68 (0.44-1.06)
	2007	1.04 (0.97-1.12)	**1.07 (1.00-1.13)**	**1.04 (1.00-1.09)**	1.46 (0.73-2.91)	0.60 (0.35-1.03)	0.85 (0.56-1.28)
	2008	**1.10 (1.03-1.18)**	**1.08(1.02-1.14)**	**1.08 (1.03-1.12)**	1.29 (0.65-2.55)	1.06 (0.67-1.69)	1.12 (0.76-1.66)

Calculated using multivariate Poisson regression Dependent variable: "Incidence of hospitalizations for RTHA".

† Calculated using logistic regression. Dependent variable: "In hospital death after hospitalizations for RTHA"

The independent variables included in the models are those shown in the table.

With regard to IHM, after adjusting the logistic regression model, controlling by age and CCI, men and women showed no significant changes in the risk of death after RTHA from 2001 to 2008.

For both sexes the risk of IHM was higher as age and CCI increased.

## Discussion

In this population based study involving in32, 280 cases, we found a small but significant increase in the incidence of RTHA from 2001 to 2008 in the Spanish population, from 20.2 to 21.1 procedures per 100, 000 inhabitants (p < 0.01). This represents a 3.75% overall increase, which is similar to other reports in developed countries [1].

In this period a total of 161, 791 discharges of patients having undergone primary total hip arthroplasty were identified and the overall crude incidence increased from 99 to 105 THA per 100, 000 inhabitants (p < 0.001) [19].

Kurtz and Mowat analyzed the National Hospital Discharge Survey (NHDS) of the USA, 1990 through 2002, to study the changes in the revision burden. The rate of RTHA increased by 3.7 procedures per 100, 000 persons over a 10 year period [1].

In the United States and Canada the revision burden, which refers to the percentage of revision hip replacements relative to the total number of primary and revision hip replacements, stayed roughly the same in the USA (14%-17% from 1993 to 2005) and in Canada (11%-13% from 2001 to 2006) [6], reflecting an increase in the absolute number of revisions as the number of primary procedures also increased.

This same trend is observed in some National Registries. In Norway the revisions constituted 12.3% of all the operations in 2003, 13.6% in 2007 and 14% in 2008 [20].

In 2009, the number of hip replacements reported to the Australian Registry increased by 1, 100 (3.4%) compared to 2008. Primary THA increased by 4.0% and RTHA by 1.1%. From 2003 to 2009 primary total hip replacement increased by 32.5% and revision hip replacement 9.3%. Remarkably, revision hip replacement as a proportion of all hip replacement procedures declined from 13.0% in 2003 to 11.2% in 2009 [21].

In Spain the ratio RTHA/THA has remained stable with figures around 20% from 2001 to 2008. The equivalent percentages for the Norwegian Arthroplasty Registry were 14.94% (922/6170) in 2001 and 16.37% (1114/6804) in 2008 [20].In Australia in 2004 the ratio was19.25% (3494/18153) decreasing to 16.63% (3677/22109) in 2008 [21]

In our study the results of the Poisson regression analysis confirm that the increase in incidence of RTHA in men and also in women became greater after adjusting for potential confounders (age, sex and CCI). Although all groups increased in incidence those patients aged 75-84 or 65-74 experienced the highest (13.9 and 9.5 with 95% interval confidence of 13.3-14.5 and 9.1-9.9, respectively). Other studies using multivariate models have reached the same conclusions, verifying that the increase in incidence of RTHA is not only a consequence of population growth or ageing [3,4,22].

In a recent study Cram et al, in an observational cohort of 1, 453, 493 Medicare beneficiaries who underwent THA between 1991 and 2008 in the USA, observed that the mean age for patients increased from 74.1 to 75.1 years (P < 0.001). [23]. In that same population and time period, among 348 596 subjects who underwent RTHA, the mean age also raised from 75.8 to 77.3 years (p < 0.001). [23].

In Spain, the number of high-risk surgical patients has increased over the last 8 years as shown by the analysis of the CCI. In 2001, 19% of patients had a Charlson Index of 1-2 or > 2. In 2008, the proportion of patients who had undergone a RTHA and had Charlson Index of 1-2 or > 2 had increased to 24.6% (p < 0.001). This same trend has been previously described in other studies and in the present series in the same period in THA [12,19,24].

A higher severity of illness store has been reported as predictive of a higher resource utilization for both primary and revision arthroplasty [25].

Our co-morbidity index figures for primary THA and RTHA are surprisingly similar. Cram et al [23] reported a significant mean increase in the number of comorbid illnesses per patient (from 1.0 to 2.0 for THA and 1.1 to 2.3 for RTHA) [23]. The similar morbidity index observed in our patients undergoing primary and revision THA could be explained by survival (only healthier subjects survive long enough to need a revision) and selection (surgeons only conduct RTHA among those patients with low co-morbidities) bias. Further studies should be conducted to verify this.

LOS decreased from an average of 20.6 days in 2001 to 19.1 days in 2008 in Spain (p < 0.01), which is a small but relevant change.

In the same period, the LOS for THA significantly decreased from an average of 13 days in 2001 to 10.45 days in 2008 [19].

The mean nationwide LOS for Spain is longer than that described in other countries although a wide variability has been reported [23,26].

As also suggested by other authors, we hypothesize that the reasons for observing a decrease in the Spanish LOS overtime may include: the presence of a larger rate of specialized departments using more efficient means to rehabilitate and discharge patients and an increased rate of discharges to short and long-term care facilities [26-28]. After adjusting by age and sex, our rate of patients that were discharge to health or social institutions significantly rose from 4% to 6.4% from 2001 to 2008.

The total costs of RTHA in Spain during our study period increased by 114.4%, from 34.8 million Euros to 74.6 million Euros. After adjusting for inflation, the average costs per patient increased by 78.3%, from 9, 375 to 16, 715 Euros. The total costs of primary THA In Spain during this period increased by 75%, from 120.6 million Euros to 211.34 million Euros [19].

Stargardt studied the variations in the cost of THA between and within nine member states of the European Union (EU), including Spain. The main cost drivers were found to be implants (34% of total cost on average) and ward costs (20.9% of total cost on average) [29].

More complicated revisions, like those requiring bone grafting, exchange of both components or specific complications, like periprosthetic fractures or infections, require higher resource utilization than easier ones [25,30-33]. Even if we don't have data detailing the reason for revision we believe that the use of specialised 'revision-implants' is in part responsible of the large increase in the average cost per patient overtime in Spain.

The overall IHM after RTHA in Spain ranged from 1.16% in 2001 to 1.77% (p: 0.025) in 2008 (1.40% in women and 0.80% in men in 2001 and 2.02% in women and 1.44% in men in 2008).

Zhan and Kaczmarek screened the hospital discharge abstracts National Hospital Discharge Survey (NHDS) of five states of the EU during the year 2003. Their reported IHM rate was 0.84% and their rate of readmission, for any cause, within thirty days was 8.48%. Advanced age and co-morbid diseases were associated with worse outcomes [34].

In RTHA, prognostic factors related to higher mortality rates or complications may not be as clearly stated as in THA. Older age and high CCI may be more consistent but others like complexity of the revision, infected RTHA, poor preoperative functional status or female sex also seem to be important. These prognostic factors should help to optimize indications for THA and to reduce the already staggering, yet growing, burden of RTHA in developed countries, compared to THA, with greater LOS and higher cost [11,22,30,35-38].

To the best of our knowledge this is the first study in Spain regarding changes in the incidence, demographic characteristics, co-morbidity profiles, and in-hospital outcomes of patients undergoing RTHA. In the absence of a National Registry for Arthroplasty the incidence, IHM rates and cost estimation reported in this study provide the best available information. The main strength of the current study lies in a large sample size and standardized methodology maintained over the study period.

Nevertheless, the present study presents some limitations. First, a potential source of bias comes from relying on administrative registries as several discrepancies between administrative data and audited and validated clinical data have been suggested [39,40].

In Spain the CNBD was implemented in 1996 by the Ministry of Health in liaison with all of the autonomous communities. From that time efforts were made to improve the quality of the information including: periodic publications; mandatory educational programs for the persons responsible for the codification in the hospitals and periodical external quality control audits. Previous Spanish studies have assessed the validity of CMBD data using medical records as a reference, reporting that the CMBD is reliable for diagnosis and for estimating adjusted mortality rates. [41,42].

Second, even if administrative data generally agrees with patient chart data for recording of comorbidities, it has been found that comorbidities tend to be under-reported in administrative data [43-45].

This could explain the large healthy population found in our study, based on our CCI. With regard to the use of the Charlson index to measure comorbidities, *Burgos E et al conducted an investigation to assess the predictive value of six functional status and/or surgical risk scoring systems, including the anesthesiologic risk assessment instrument (ASA) and the Charlson index, with regard to serious complications after hip fracture surgery in the elderly. They found that all the scoring system reached a sufficient predictive value with regard to serious post-operative complications *[46]. However the similarities and differences between the Charlson comorbidity index and other commonly use score such as the ASA classification are discussed by Weaver et al. These authors found that among patient undergoing joint arthroplasties the discrepancy between the comorbidity index and the ASA was striking [47].

Lastly, outcomes were limited to the variables coded. The lack of differentiation between types of RTHA procedures in the current ICD-9-CM procedure coding system limits the utility of these codes in evaluating differences in patient and procedure characteristics in large public data. Other confounding relevant variables such as surgeon, hospital volume, cause leading to revision or the percentage of one-stage and two-stage revisions could not be analyzed as these variables are not collected in the database This lack of information about different brands thus unable to pick-up specific implant-related failures, compared with the National Joint Registries [20,21].

Therefore, outcomes such as LOS and discharge destination may have been influenced by other covariates different from postoperative complications. In such a scenario, only IHM or LOS can be used to draw direct conclusions on the complication rate in the current study.

As noted above, because we used anonymised data it is impossible to detect double registrations, readmissions and transfers using the CMBD. Furthermore, unfortunately, in Spain we have no estimation of the number of double registrations, readmissions and transfers among patients undergoing a hip arthroplasty so a correction factor including this information could not be used to adjust the data. This is relevant because previous studies conducted in other countries have shown that the number of readmissions and transfers may have changed over time and may differ with age and sex [48,49].

We believe that, notwithstanding its limitations the CMBD is a valid instrument to conduct epidemiological studies and has previously been used for this purpose, including by other authors. [20,50,51].

Although DRG have been a useful patient classification system for hospital cost analysis, DRG present a series of limitations [52,53]. Riley in a recent review concludes that because administrative data have been collected for other purposes it is therefore not necessarily in a format that is intelligible or convenient to researchers. Furthermore, coding of diagnoses and procedures are more closely related to billing requirements than to medical records.

## Conclusions

In conclusion the present study indicates a small but significant increase in the crude and adjusted incidence of RTHA in Spain from 2001 to 2008 in line with results observed in other countries. We also found reductions in LOS with a significant increase in cost per patient. The health profile of the patient undergoing RTHA seems to be worsening in Spain. These results may reflect broadening of the indication criteria for these procedures in conjunction with the natural failures of THA implanted in the previous decades.

This time trend may be useful for planning future resources and to optimize indications and proper patient selection.

## Abbreviations

CCI: Charlson comorbidity index; DRGs: Diagnosis-Related Groups; IHM: In-hospital mortality; LOS: length of hospital stay; THA: Total hip arthroplasty; RTHA: Revision Total hip arthroplasty.; CMBD: Spanish National Hospital Database namely Conjunto Minimo Basico de Datos.

## Competing interests

The authors declare that they have no competing interests.

## Authors' contributions

MVM - Conception and design, acquisition of data, interpretation of data and drafting the manuscript. VHB -Acquisition of data, analysis and interpretation of data and drafting the manuscript. ARL- Analysis and interpretation of data and drafting the manuscript. FCR - Analysis and interpretation of data and drafting the manuscript. JRM- Analysis and interpretation of data and drafting the manuscript. JSRM - Analysis and interpretation of data and drafting the manuscript. AGDM - Conception and design and revising the manuscript critically for important intellectual content; RJG - Conception and design, acquisition of data, analysis and interpretation of data and drafting the manuscript. All authors have given final approval of the version to be published.

## Pre-publication history

The pre-publication history for this paper can be accessed here:

http://www.biomedcentral.com/1471-2474/13/37/prepub
